# The Dynamic Expression Changes of Neutrophil Extracellular Traps in Mouse Apical Periodontitis: A Potential Correlation With IL‐17

**DOI:** 10.1155/jimr/8039031

**Published:** 2025-09-15

**Authors:** Zihan Ma, Xiaoyue Sun, Ying Lin, Zijun Wang, Qing Nie, Jingjing Yu, Jingwen Yang, Lingxin Zhu

**Affiliations:** ^1^ State Key Laboratory of Oral and Maxillofacial Reconstruction and Regeneration, Key Laboratory of Oral Biomedicine Ministry of Education, Hubei Key Laboratory of Stomatology, School and Hospital of Stomatology, Wuhan University, Wuhan, 430079, China, whu.edu.cn; ^2^ Department of Stomatology, Shenzhen Children’s Hospital, Shenzhen, 518026, China, szkid.com.cn

**Keywords:** apical periodontitis, bone destruction, IL-17, neutrophil extracellular traps, osteoclast

## Abstract

Neutrophil extracellular traps (NETs) consist of decondensed chromatin and antimicrobial proteins, which are released from neutrophils and have been implicated in several inflammatory diseases. NETs and interleukin (IL)‐17 constitute a feed‐forward loop that promotes immunopathological development of inflammation. Therefore, this study aimed to investigate the dynamic distribution of NETs and their colocalization with IL‐17 during the progression of apical periodontitis in established mouse models. Apical periodontitis was induced in mice by exposing the pulp of the mandibular first molars, with mandibles harvested on day 0, 7, 14, 21, and 28 after pulp exposure. Micro‐CT and high‐resolution X‐ray scanning showed progressive increases in both the area and volume of periapical lesions from day 0 to day 28. Osteoclasts in the periapical lesions were identified using tartrate‐resistant acid phosphatase (TRAP) staining, with their numbers peaking on day 21. Immunofluorescence staining was performed for citrullinated histone H3 (CitH3), myeloperoxidase (MPO), neutrophil elastase (NE), and IL‐17 to localize NETs and their colocalization with IL‐17 in lesions. NETs, which were visible on day 7 and increased gradually until day 21, primarily located in inflammatory infiltration areas of the periapical tissue. Additionally, western blot analysis showed increased CitH3 expression in periapical lesions on day 21 after pulp exposure, further confirming the presence of NETs. Both RT‐qPCR and enzyme‐linked immunosorbent assay (ELISA) revealed increased IL‐17 expression in periapical lesions. The colocalization of CitH3, the major component of NETs and IL‐17 peaked on day 21 after pulp exposure. Furthermore, in vitro experiments demonstrated that IL‐17 promoted NETs formation under lipopolysaccharide (LPS)‐simulated inflammatory conditions. Our findings indicated that NETs expression changed dynamically and suggested a feedback loop between NETs and IL‐17 during the development of apical periodontitis in mouse models.

## 1. Introduction

Apical periodontitis is a common oral inflammatory disease characterized by both microbial infection of the dental pulp and the resulting host immune response, leading to inflammation and bone destruction in the periapical tissue [1]. Periapical tissue inflammation induces the secretion of various cytokines, including interleukin (IL)‐1, IL‐6, IL‐17, TNF‐alpha(α), and INF‐gamma(γ) [2]. Our research group found that IL‐17 and its primary source, Th17 cells, are closely related to the pathogenesis of periapical diseases [3–5]. The local production of IL‐17 is associated with neutrophil infiltration and the release of neutrophil extracellular traps (NETs) in inflammatory microenvironment [6]. However, whether there exists a regulatory relationship between NETs and IL‐17 in periapical lesions remains unknown.

NETs are web‐like extracellular structures composed of decondensed chromatin decorated with cytosolic and granular proteins, representing a novel mode of neutrophil‐mediated immunity [7]. Upon stimulation by microorganisms or sterile inflammation, NETs are released primarily through a cell death process, known as neutrophil extracellular trap formation (NETosis) [8]. On one hand, NETs protect against infection, particularly by large microbial pathogens; on the other hand, they are also implicated in the pathogenesis of immune‐mediated disease [7]. During NETosis, intracellular autoantigens and enzymes are released directly to damage host tissues, contributing to the pathogenesis of diseases, including sepsis, atherosclerosis, autoimmunity, and periodontitis [9–12]. In the early stage of murine apical periodontitis (day 7 after pulp exposure), NETs were detected in the periapical tissues [13]. However, whether NETs persist during the chronic phase of apical periodontitis, as well as, their functional interplay with cytokines remain unknown.

Accumulating evidence from human and animal studies demonstrates that IL‐17, a pro‐inflammatory cytokine, is present in apical periodontitis and likely contributes to periapical bone destruction [3–5]. IL‐17 facilitates neutrophil mobilization and NET release in various inflammatory diseases [14, 15]. Moreover, NETs stimulate T cell activation via NET‐associated histones and specifically drive Th17 cell differentiation, amplifying IL‐17 production [10–12]. This feed‐forward loop between NETs and IL‐17 may thus exacerbate the immunopathological development of inflammatory diseases. Nevertheless, the potential relationship between NETs and IL‐17 in apical periodontitis remains unexplored. Therefore, this study aimed to investigate the occurrence and dynamic changes of NETs in the established mouse model of apical periodontitis, and examine their relationship with IL‐17.

## 2. Materials and Methods

### 2.1. Animals

A total of 30 C57BL/6J male mice aged 7–8 weeks and weighing about 20 g were obtained from the Experimental Animal Center of Hubei Province, China. All mice were housed in a specific pathogen free (SPF) facility under a 12 h light/dark cycle, with ad libitum access to standard rodent chow diet. Pulp exposure procedures were performed under sodium pentobarbital anesthesia (50 mg/kg), and all handling was conducted by trained personnel using gentle techniques to minimize stress. All animal experiments were approved by the Animal Research Ethics Committee of Wuhan University, China (Approval Number WP20210535).

### 2.2. Induction of Apical Periodontitis and Sample Preparation

The mouse model of apical periodontitis was established as previously described [16]. Briefly, the mice (*n* = 6) were anesthetized by intraperitoneal injection of sodium pentobarbital anesthesia (50 mg/kg). Pulp exposure of the mandibular first molars were performed using a #1/4 round burr, and were left open in the oral environment without any coverage. On days 0, 7, 14, 21, and 28 after pulp exposure, six mice from each group were euthanized via cervical dislocation by a trained professional, and the mandibles were subsequently collected. Samples were fixed in 4% paraformaldehyde at 4°C for 48 h, then decalcified in 10% EDTA solution. After decalcification and dehydration, the mandibles were embedded in paraffin and then sectioned into 5 μm slices.

### 2.3. High‐Resolution X‐Ray Imaging and Micro‐CT Analysis

High‐resolution X‐ray images were taken through DXS PRO (Bruker Corporation, USA) and analyzed using Bruker Molecular Imaging software to observe bone density of periapical lesions in mice as previously described [16]. Micro‐CT scanning was performed on the mandibles (SkyScan 1276; Bruker microCT, Belgium). The reconstructing image data was obtained using the NRecon software and measured with CT Analyzer and Dataviewer software [16]. An investigator captured Micro‐CT and X‐ray images under standardized parameter conditions. Additionally, two researchers independently performed blinded measurements of lesion size for calibration purposes.

### 2.4. Histological Examination

Sequential paraffin sections that clearly showed the distal root apex of the mandibular first molar, as well as, the central part of the pulp and root canal, were selected for staining and morphometric analysis of the periapical lesion area and volume. After deparaffinization, sections were rehydrated and stained with hematoxylin and eosin (H&E). One of every four sections was observed under light and fluorescence microscope (Leica, Germany).

### 2.5. Tartrate‐Resistant Acid Phosphatase (TRAP) Histochemical Staining

TRAP staining was performed using a commercial kit (Sigma, USA; catalog number 387A) according to the manufacturer’s protocol to identify osteoclasts. Deparaffinized and rehydrated sections were sequentially incubated in Naphthol AS‐BI phosphate solution, freshly prepared Fast Garnet GBC solution for 1 h at 37°C, then stained with hematoxylin. Substrate‐negative controls were processed identically but without Naphthol AS‐BI phosphate. Multinuclear (≥ 3 nuclei) cells exhibiting dark red/purple cytoplasmic staining were defined as osteoclasts. For quantification, TRAP‐positive cells were counted in three randomly selected periapical areas per distal root under 40 × objective magnification [16].

### 2.6. Immunofluorescence Labeling

The following experimental steps were followed for all paraffin sections; dewaxing in xylene, passing through a gradient ethanol solution into water, followed by incubation with pepsin (Maixin, China; catalog number DIG‐3009) for 30 min at 37°C. The sections were then incubated with 2.5% bovine serum albumin (BSA) for 1 h at 37°C to block the endogenous peroxidase activity. Without washing, the sections were incubated with anti citrullinated histone H3 (CitH3) rabbit antibody (Abcam, UK; catalog number ab5103; 1:200), myeloperoxidase (MPO) goat antibody (R&D Systems, USA; catalog number AF3667; 1:100), IL‐17 mouse monoclonal antibody (Santa Cruz Biotechnology, USA; catalog number sc‐374218; 1:50), or neutrophil elastase (NE) rabbit antibody (EPITOMICS, China; catalog number 5953‐S, 1:100) at 4°C overnight. After washing the sections thoroughly, they were incubated with donkey anti‐goat IgG (Dylight 488‐conjugated, Abbkine, China; catalog number A24231), goat anti‐mouse IgG (Dylight 488‐conjugated, Abbkine, China; catalog number A23210) or goat anti‐rabbit IgG (Dylight 594‐conjugated, Abbkine, China; catalog number A23420) at 37°C for 60 min in darkness. Subsequently, the sections were infiltrated with mounting medium containing DAPI (Zhongshan, China; catalog number ZLI‐9600) and observed under a fluorescence microscope (Leica, Germany). Negative controls were incubated with nonimmune bovine serum as the primary antibody. All images were taken under identical exposure settings. Quantification of immunofluorescence labeling was performed as previously described [17, 18]. Each sample was randomly divided into three fields and analyzed. Subsequently, the mean fluorescence intensity of the labeled protein was analyzed by ImageJ software and the Pearson correlation coefficient was calculated to quantify the colocalization between the foci.

### 2.7. Western Blot Analysis

The mandibular first molars, along with a section of bone, were homogenized in ice‐cold RIPA lysis buffer (Beyotime, China; catalog number P0013B) supplemented with protease and phosphatase inhibitor cocktail (MedChemExpress, USA; catalog number HY‐K0010, HY‐K0021). The homogenate was centrifuged (12,000 × *g*, 15 min, 4°C) to collect protein supernatants. Protein concentrations were quantified using the BCA Protein Assay Kit (Beyotime, China; catalog number P0012). Equal amounts of protein (10 μg) were loaded onto SDS‐PAGE gels, and transferred to PVDF membranes (Roche, Germany; catalog number 03010040001), then the membranes were blocked with 5% (w/v) milk for 1.5 h. The membranes were then incubated overnight at 4°C with the primary antibodies to CitH3 (Abcam, UK; catalog number ab5103; 1:1000), β‐actin (Proteintech, USA; catalog number 66,009‐1; 1:15,000), followed by incubation with HRP‐conjugated anti‐rabbit secondary antibodies (Cell Signaling Technology, USA; catalog number 7075S) and anti‐mouse secondary antibodies (Cell Signaling Technology, USA; catalog number 7076S) for 1 h.

### 2.8. RNA Extraction and RT‐qPCR Analysis

The mandibular first molars, along with a section of bone, were triturated in liquid nitrogen. Total RNA was isolated with TRIzol reagent (Invitrogen, USA; catalog number. 15596026). cDNA was then synthesized using the reverse transcription reaction kit (Vazyme, China; catalog number R223‐01). Gene expression was quantitated using SYBR qPCR Master Mix (Vazyme, China; catalog number Q312‐02) and the mouse primers on a CFX384PCR system (Roche, Germany). All primer sequences were shown in Table 1.

**Table 1:** The primers used in this study.

Gene	Sequence (5′–3′)	Length (bp)	Annealing temperatures (°C)
IL‐17a	5′‐CAGACTACCTCAACCGTTCCAC‐3′ 5′‐TCCAGCTTTCCCTCCGCATTGA‐3′	130	55
TNF‐α	5′‐CAGGCGGTGCCTATGTCTC‐3′ 5′‐CGATCACCCCGAAGTTCAGTAG‐3′	89	55
IL‐6	5′‐TAGTCCTTCCTACCCCAATTTCC‐3′ 5′‐TTGGTCCTTAGCCACTCCTTC‐3′	76	55
β‐Actin	5′‐GTGACGTTGACATCCGTAAAGA‐3′ 5′‐GCCGGACTCATCGTACTCC‐3′	245	55

### 2.9. Enzyme‐Linked Immunosorbent Assay (ELISA)

Periapical tissue from the mandibular first molars along with surrounding bone, were carefully excised and weighed. The samples were then homogenized in 300 μL ice‐cold PBS, supplemented with protease inhibitor cocktail. After centrifugation (12,000 × *g*, 15 min, 4°C), supernatants were collected for analysis. IL‐17 concentrations in tissue lysate were measured using the IL‐17 ELISA kit (NEOBIOSCIENCE, China; catalog number EMC008) following the manufacturer’s guidelines.

### 2.10. Mouse Neutrophil Isolation

Neutrophils were extracted following the manufacturer’s guidelines (TBD Science, China; catalog number TBD2013NM). Briefly, neutrophils were collected from femurs and tibias of 8‐week‐old C57BL/6J mice, and cultured in Dulbecco’s modified eagle medium (Gibco, USA; catalog number 11965092) supplemented with 10% heat‐inactivated FBS (Sigma, USA; catalog number F2442). The cells were treated with 100 ng/mL lipopolysaccharide (LPS, Sigma, USA; catalog number LPS25), with or without 10 ng/mL IL‐17 (MedChemExpress, USA; catalog number HY‐P73174) for 4 h.

### 2.11. Cellular Immunofluorescence Staining

Cells were washed with PBS, fixed with 4% paraformaldehyde and permeabilized with 0.1% Triton X‐100 for 15 min. The cells were washed with PBS, blocked with goat serum for 1 h at 37°C, and subsequently incubated overnight at 4°C with a mixture of two primary antibodies: CitH3 antibody (Abcam, UK; catalog number ab5103; 1:200) and MPO antibody (R&D Systems, USA; catalog number AF3667; 1:100). The cells were stained with secondary fluorescent antibodies, mounted with DAPI, and then observed using a confocal microscope (Olympus SpinSR, Japan). All images were taken under identical exposure settings. Mean fluorescence intensity was quantified using ImageJ software.

### 2.12. Statistical Analysis

Data are presented as mean ± standard deviation (SD). All data were analyzed using the Shapiro–Wilk test to confirm that they followed a normal distribution. Significant differences between the two groups were analyzed using an unpaired two‐sided Student’s *t*‐test, while differences between multiple groups were analyzed using one‐way analysis of variance (ANOVA) and Tukey’s multiple comparison test in GraphPad Prism 7.0 (GraphPad Software Inc.). *p*‐Value < 0.05 was considered significantly different.

## 3. Results

### 3.1. Morphometric Analysis of the Area of the Periapical Lesions

Micro‐CT measurements showed a progressive increase in lesion volume and area from day 0–28 (Figure 1A). Consistent with these findings, high‐resolution X‐ray imaging revealed expanding bone destruction in periapical tissues over time (Figure 1A). Notably, the largest increase in bone resorption occurred from day 7–14, with lesion volume showing an approximate 0.1 mm^3^ increase and lesion area expanding by roughly 0.1 mm^2^ (Figure 1B,C). Histological analysis revealed few inflammatory cells in the periapical tissue on day 0, with significantly increased inflammatory cell infiltration over time after pulp exposure (Figure 2A,B). Inflammatory cell infiltration in lesions was accompanied by osteoclast formation and bone resorption. Osteoclasts were absent on day 0, and their number gradually increased from day 7–21 but decreased from day 21–28 (Figure 2C,D). The results suggested that periapical inflammation transition from the acute stage to the chronic stage from day 21–28 after pulp exposure, entering a stable phase characterized by a reduction in osteoclast numbers.

Figure 1Assessment of bone destruction in mouse periapical lesions by high‐resolution X‐ray and Micro‐CT. (A) High‐resolution X‐ray and Micro‐CT evaluations of lesion size and bone destruction range of mouse apical periodontitis. Representative Micro‐CT images in two‐dimensional, including the sagittal (Sag), coronal (Cor), and horizontal (Hor) directions, and periapical lesions, are marked in red. (B) Quantitative analysis of the periapical lesion area in mice with apical periodontitis at each time point. *n* = 6. (C) Quantitative analysis of the periapical lesion volume in mice with apical periodontitis at each time point. *n* = 6.  ^∗^ *p* < 0.05,  ^∗∗^ *p* < 0.01,  ^∗∗∗^ *p* < 0.001, and  ^∗∗∗∗^ *p* < 0.0001. The statistically significant *p*‐value was set at *p* < 0.05.

**Figure:**
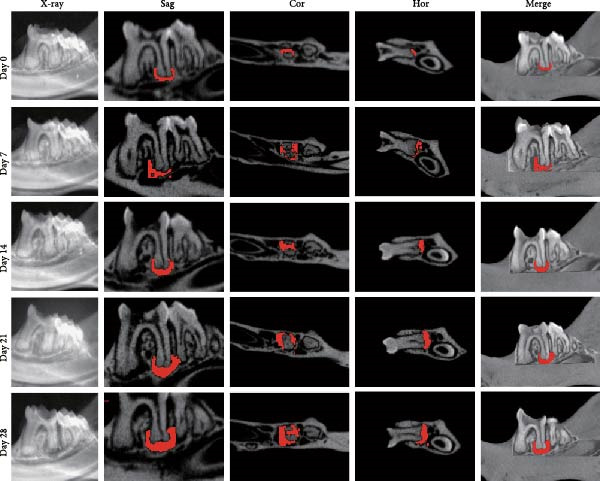
(A)

**Figure:**
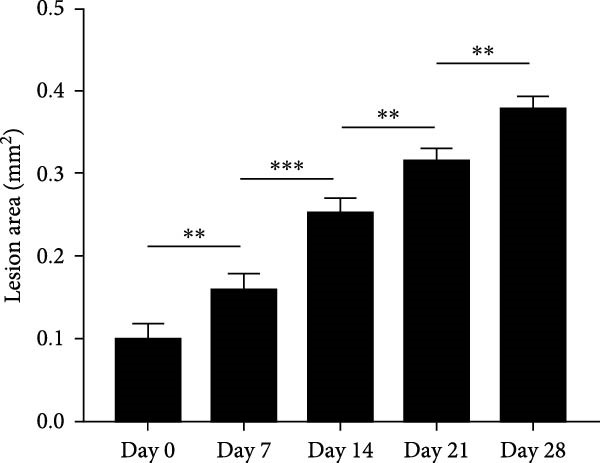
(B)

**Figure:**
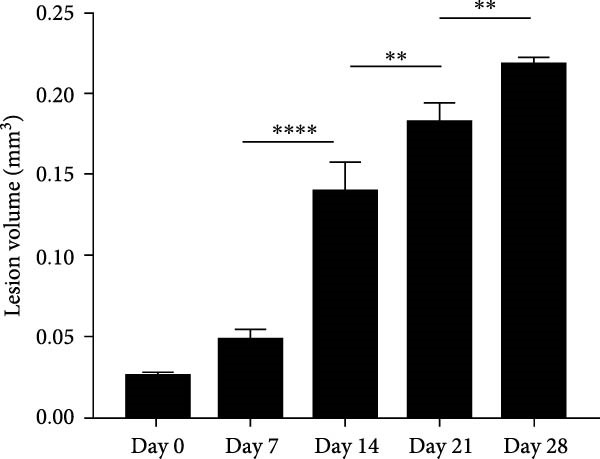
(C)

Figure 2Histological evaluation of periapical lesion size and inflammatory cell infiltration in mice. (A) Representative images of H&E staining for mouse apical periodontitis. Bar indicates 100 μm. (B) Quantitative analysis of the periapical lesion area in mice with apical periodontitis using H&E staining. *n* = 6. (C) Representative images of TRAP staining for mouse apical periodontitis. The black arrows indicated TRAP‐positive cells. Bar indicates 100 μm. (D) Quantitative analysis of osteoclasts in mice with apical periodontitis with TRAP staining. *n* = 6.  ^∗^ *p* < 0.05,  ^∗∗^ *p* < 0.01,  ^∗∗∗^ *p* < 0.001, and  ^∗∗∗∗^ *p* < 0.0001. The statistically significant *p*‐value was set at *p* < 0.05.

**Figure:**
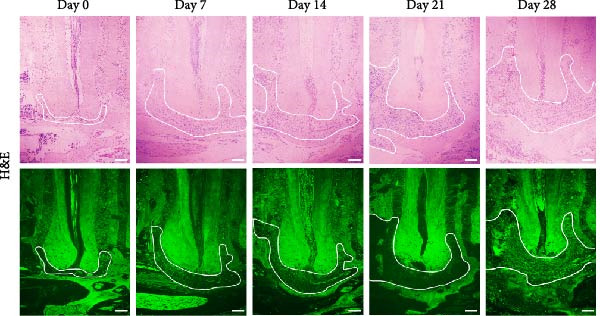
(A)

**Figure:**
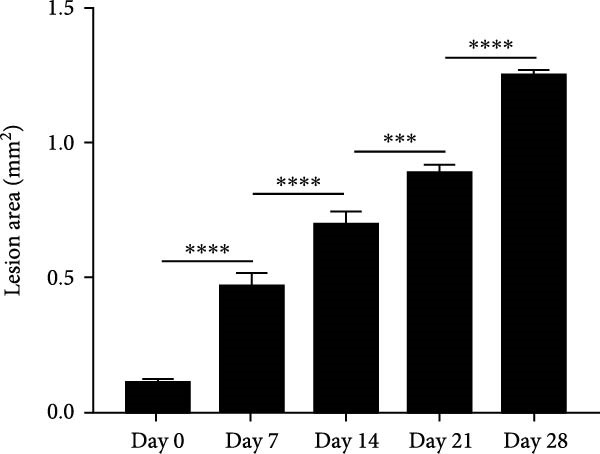
(B)

**Figure:**
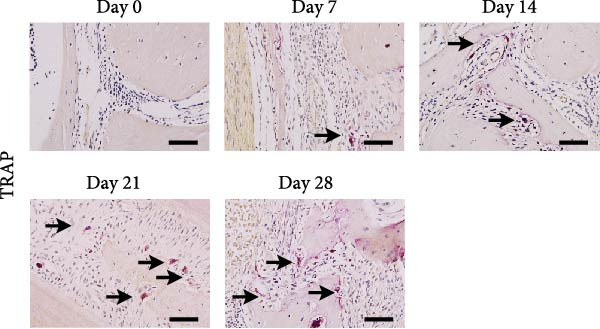
(C)

**Figure:**
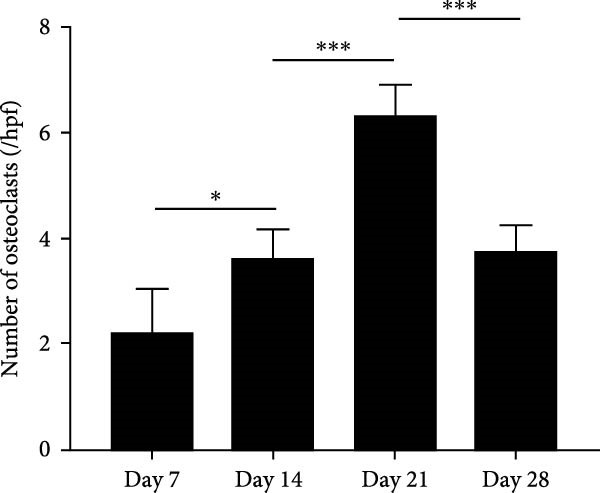
(D)

### 3.2. NETs Changed Dynamically in Periapical Lesions at Different Stages

NETs in periapical lesions were identified by colocalization of CitH3, MPO, and DNA through immunofluorescent staining. In the Figure 3A, red staining represented CitH3, green staining indicated MPO, and blue DAPI labeled DNA. The areas depicted in yellow were considered to be the NETs, which were the positive areas superimposed by CitH3 and MPO (Figure 3A). Only minimal colocalization of CitH3 and MPO was detected in the non‐inflamed (day 0) periapical areas, while clear and increased colocalization was observed in inflammatory cell‐infiltrated lesions on day 7 after pulp exposure (Figure 3B–D). Quantitative analysis demonstrated a progressive increase in NETs formation from day 7–21, peaking at day 21, followed by a significant reduction by day 28 (Figure 3D). These findings indicate active NETs formation during the progression of apical periodontitis, with its dynamic formation patterns correlating with inflammatory stage transitions. Additionally, NE is also one of the key components of NETs, contributing to the stability of NETs structure and playing a role in its antimicrobial function [19]. The immunofluorescence staining results revealed that the expression of NE was highest on day 21 (Figure S1A,B). Western blot analysis revealed that the expression of CitH3 in periapical lesions was upregulated on day 21 compared to day 0 after pulp exposure (Figure S1C). The above results further confirmed that the expression of NETs increased during the development of apical periodontitis.

Figure 3Dynamic changes in NETs in mouse periapical lesions. (A) Representative images of CitH3 (red), MPO (green), and DAPI (blue) immunolabeling of periapical lesions. The selected boxes provided a partial enlarged view of the NETs in the stained images. The white arrows indicated the coexpression regions. Bar indicates 100 μm. (B) Quantitative analysis of mean fluorescence intensity of CitH3. *n* = 6. (C) Quantitative analysis of mean fluorescence intensity of MPO. *n* = 6. (D) Quantitative analysis of the colocalization of CitH3 and MPO using Pearson’s coefficient. *n* = 6.  ^∗^ *p* < 0.05,  ^∗∗^ *p* < 0.01,  ^∗∗∗^ *p* < 0.001, and  ^∗∗∗∗^ *p* < 0.0001. The statistically significant *p*‐value was set at *p* < 0.05.

**Figure:**
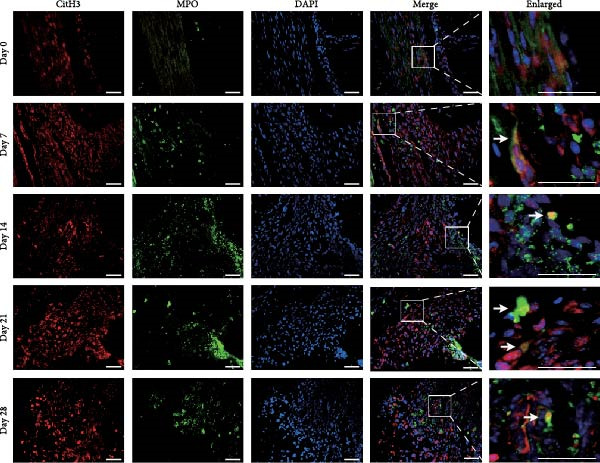
(A)

**Figure:**
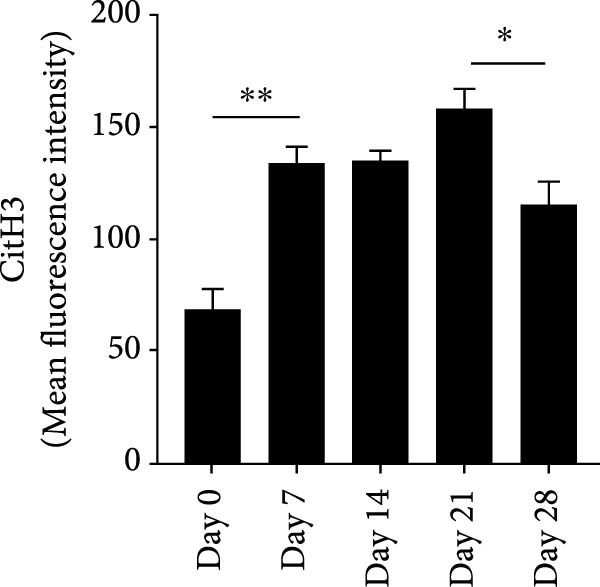
(B)

**Figure:**
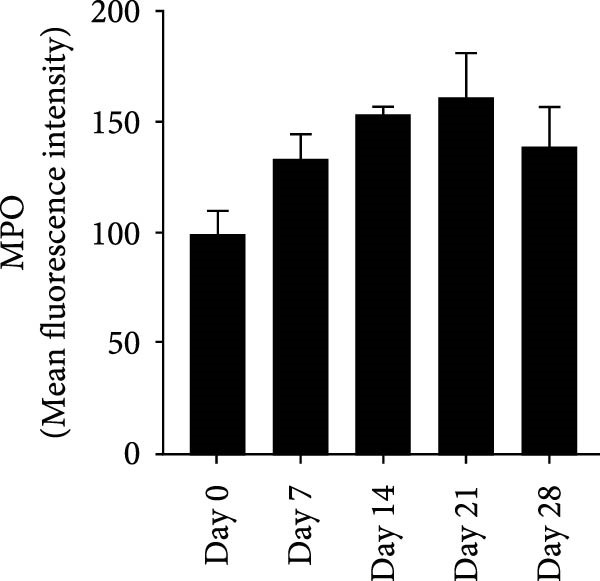
(C)

**Figure:**
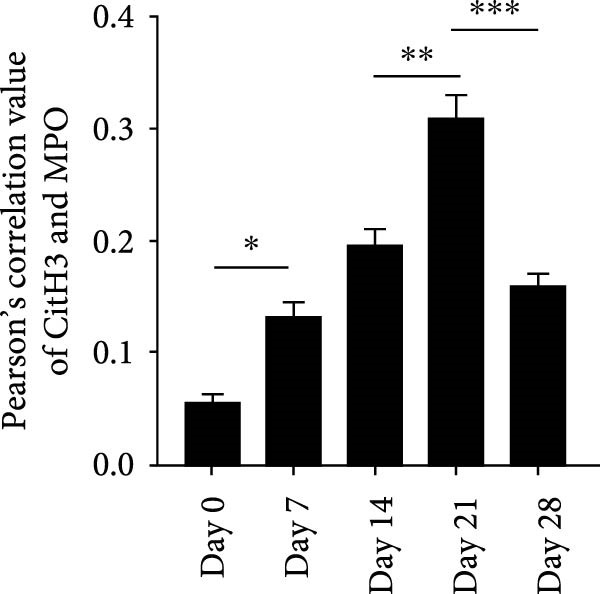
(D)

### 3.3. Colocalization of NETs With IL‐17 in Periapical Lesions

RNA extracted from periapical lesions for RT‐qPCR analysis, revealing significant upregulation of IL‐17, TNF‐α, and IL‐6 gene expression on day 21 compared to day 0 after pulp exposure (Figure S2A). ELISA quantification of IL‐17 further corroborated this finding, confirming that IL‐17 expression increased during the progression of apical periodontitis. (Figure S2B). Building on the established positive feedback loop between IL‐17 and NETs in inflammatory infectious diseases [11], particularly histone‐mediated IL‐17 activation [11, 12], we investigated their interplay in periapical lesions using immunofluorescence colocalization analysis for CitH3, IL‐17 and DNA (Figure 4A). In the Figure 4A, red, green, and blue staining indicate CitH3, IL‐17, and DNA, respectively. We observed that the expression of CitH3 and IL‐17 in periapical lesions peaked on day 21 (Figure 4B,C). CitH3 and IL‐17 double‐positive areas showed that IL‐17 may be associated with the formation of NETs in apical periodontitis (Figure 4D). Furthermore, we examined whether IL‐17 could promote NETs formation in vitro. Neutrophils were extracted from mice bone marrow and cultured with LPS to induce an inflammatory state, followed by stimulation with or without IL‐17. Immunofluorescence staining revealed that, compared to the control, LPS treatment increased NETs formation (Figure S3A). Interestingly, IL‐17 further enhanced NETs formation in the presence of LPS (Figure S3B–D).

Figure 4Colocalization of NETs with IL‐17 in mouse periapical lesions. (A) Representative images of CitH3 (red), IL‐17 (green), and DAPI (blue) immunolabeling of periapical lesions. The selected boxes are partially enlarged view of the colocalization area in stained images. The white arrows indicated the coexpression regions. Bar indicates 100 μm. (B) Quantitative analysis of mean fluorescence intensity of CitH3. *n* = 6. (C) Quantitative analysis of the mean fluorescence intensity of IL‐17. *n* = 6. (D) Quantitative analysis of the colocalization of CitH3 and IL‐17 by Pearson’s coefficient. *n* = 6.  ^∗^ *p* < 0.05,  ^∗∗^ *p* < 0.01,  ^∗∗∗^ *p* < 0.001, and  ^∗∗∗∗^ *p* < 0.0001. The statistically significant *p*‐value was set at *p* < 0.05.

**Figure:**
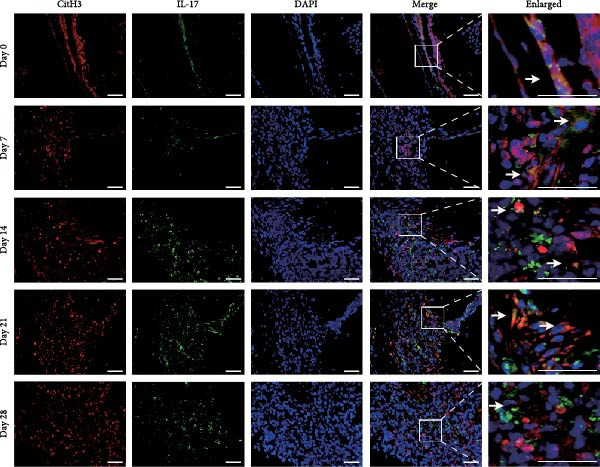
(A)

**Figure:**
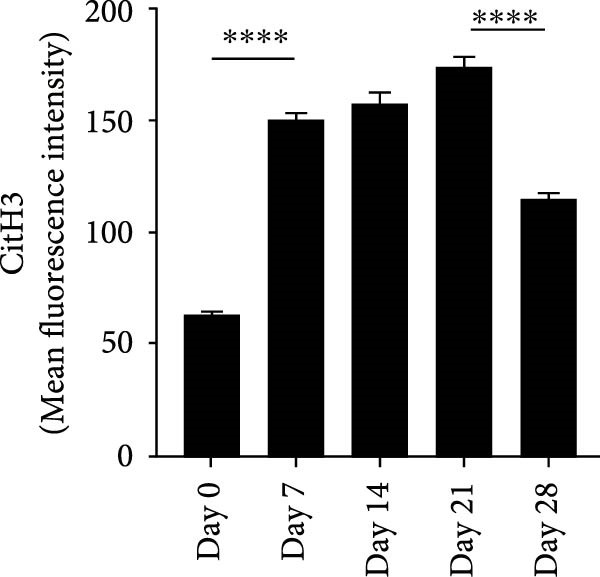
(B)

**Figure:**
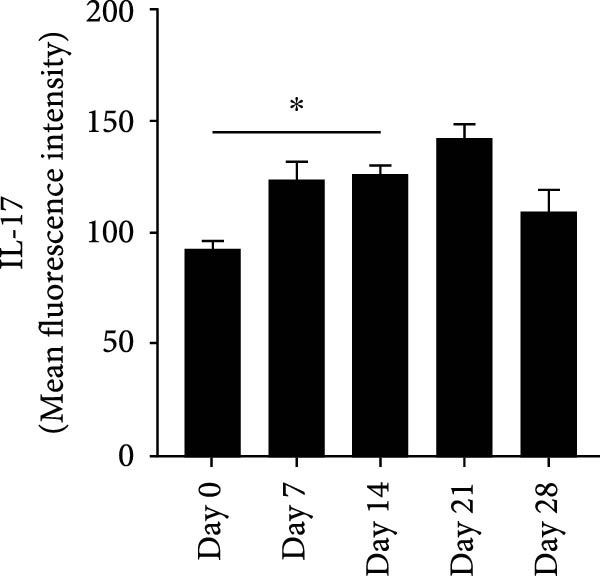
(C)

**Figure:**
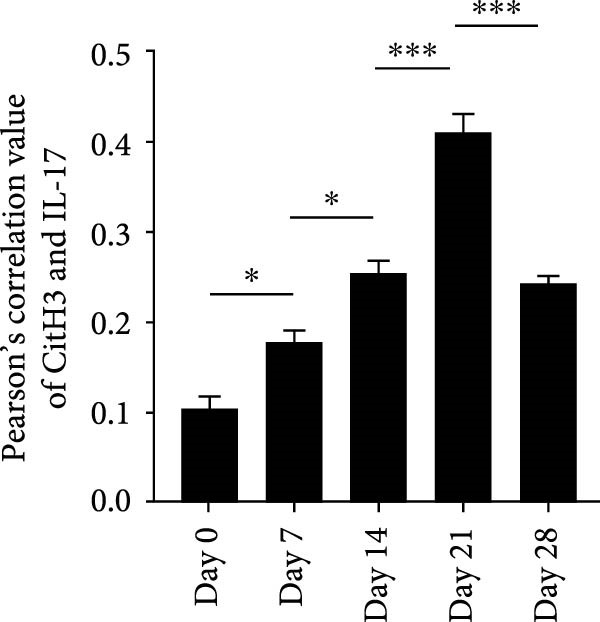
(D)

## 4. Discussion

Apical periodontitis is a prevalent inflammatory disease, affecting at least half of the worldwide adult population [20]. Although root canal therapy remains the standard clinical treatment for eliminating infection and preserving affected teeth, 2% of treated teeth are lost annually due to persistent periapical inflammation [21]. Therefore, understanding the inflammatory and immune mechanisms underlying apical periodontitis is crucial for increasing the retention time of affected teeth. This study specifically investigates the role of NETs, their dynamical changes, and potential association with IL‐17 in apical periodontitis pathogenesis.

Neutrophils serve as the first line of defense against bacterial invasion from infected root canal in necrotic and exudative periapical lesions and are thought to be an important source of inflammatory mediators that interact dynamically with chronic immune cells to promote the remission or persistence of chronic inflammation [7]. As a crucial neutrophil immune mechanism, NETs function to capture pathogens and play important roles in inflammatory diseases [7]. Recent studies have identified NETs in mouse apical periodontitis models during the acute phase (7 days postpulp exposure) [13]. Our findings revealed that NETs were present by day 7 after pulp exposure, peaked on day 21 and decreased by day 28. These temporal patterns paralleled neutrophil infiltration dynamics in periapical lesions [22], suggesting that disease progression to the chronic phase reduces neutrophil recruitment and subsequent NETs formation. In addition, the dynamic changes of osteoclasts, which may indicate that the number of osteoclasts increased continuously during the acute phase and maintained at a certain level during the chronic phase. Notably, osteoclast formation patterns correlated with NETs accumulation in periapical lesion, suggesting a potential mechanistic relationship. This observation aligns with previous findings showing reduced osteoclast numbers in periapical lesions following DNase I‐mediated NETs depletion [13].

IL‐17, a classic proinflammatory cytokine present in periapical lesions, promotes neutrophil infiltration and contributes to periapical bone destruction [14, 15]. IL‐17 recruits tumor‐associated neutrophils and induces NETosis to potentiate immunosuppressive effects in pancreatic cancer [23]. Furthermore, inhibition of histones, a major component of NETs, substantially reduces upregulation of IL‐17‐related genes (IL‐17a, S100a9, and IL‐6), decreases IL‐17‐positive cells and Th17 cells accumulation, and subsequently protects against periodontal bone loss in periodontitis [12]. Similarly, in atherosclerosis and autoimmune diseases, NETs promote Th17 cell differentiation and IL‐17 secretion [10, 11]. The identified positive feedback loop between NETs and IL‐17 led us to investigate their potential relationship in apical periodontitis. Immunofluorescence revealed colocalization of IL‐17 with the NET‐labeled molecule CitH3, suggesting functional crosstalk in apical periodontitis. Previous in vitro experiments showed that supernatants from IL‐17‐stimulated murine pancreatic adenocarcinoma cells enhances NETs formation, whereas IL‐17 alone did not directly promote the formation of NETs [23]. However, we observed that IL‐17 significantly enhanced NETs formation in the presence of LPS, suggesting that the pro‐NETs formation effect requires inflammatory priming.

In summary, our findings suggest a potential feedback loop between NETs and IL‐17 that promotes inflammatory progression and bone destruction in apical periodontitis. Treatment with DNase I, a NETs inhibitor, can inhibit NETs formation and the progression of apical periodontitis in mice [13]. Therefore, we hypothesized that the use of NETs inhibitors or anti‐IL‐17A antibodies to suppress IL‐17A may disrupt this loop, thereby alleviating apical periodontitis and bone destruction. However, our findings require further refinement, particularly regarding the use of IL‐17 knockout models to further investigate the feedback loop between NETs and IL‐17, as well as, the precise mechanisms underlying this interaction in in vivo studies of apical periodontitis. Our research provided new possibilities for exploring noninvasive treatments for apical periodontitis, while also broadening the understanding of the pathological mechanisms underlying inflammatory osteolytic diseases.

## 5. Conclusion

NETs were distributed in the inflammatory lesions of mice with apical periodontitis and changed dynamically with increasing pulp exposure time. In addition, NETs significantly correlated with IL‐17 expression and IL‐17 promoted NETs formation in the presence of LPS in vitro. Our findings highlight the critical involvement of NETs and IL‐17 in the pathogenesis of apical periodontitis and present a novel perspective on the underlying mechanisms and potential targeted therapies of inflammatory osteolytic diseases.

## Disclosure

All authors read and approved the final manuscript.

## Conflicts of Interest

The authors declare no conflicts of interest.

## Author Contributions

All authors have made substantial contributions to the conception and design of the study. Zihan Ma, Xiaoyue Sun, and Ying Lin contributed equally to this work by collecting the data, drafting the manuscript, and critically revising it. Zijun Wang and Qing Nie assisted with data analyses and data interpretation. Jingjing Yu assisted with improving the clarity and readability of the manuscript language. Jingwen Yang and Lingxin Zhu were involved in designing the study and revising the manuscript critically for important intellectual content.

## Funding

This study was funded by the National Natural Science Foundation of China (Grants 82370914, 81970919, and 82201042), the Fundamental Research Funds for the Central Universities (Grant 2042024YXA010), the Natural Science Foundation of Hubei Province (Grant 2022CFB658), and the International Science and Technology Cooperation Project of Hubei Province (Grant 2024EHA062).

## Supporting Information

Additional supporting information can be found online in the Supporting Information section.

## Supporting information


**Supporting Information** Figure S1. Dynamic changes in neutrophil elastase (NE) and CitH3 in mice periapical lesions. (A) Representative images of NE (red) and DAPI (blue) immunolabeling of periapical lesions. Bar indicates 100 μm. (B) Quantitative analysis of the mean fluorescence intensity of NE. *n* = 3. (C) Western blot for CitH3 of the periapical lesions. *n* = 3.  ^∗^ *p* < 0.05,  ^∗∗^ *p* < 0.01,  ^∗∗∗^ *p* < 0.001,  ^∗∗∗∗^ *p* < 0.0001. The statistically significant *p*‐value was set at *p* < 0.05. Figure S2. Dynamic changes in IL‐17 in mice periapical lesions. (A) RT‐qPCR analysis for IL‐17a, IL‐6, TNF‐α and (B) ELISA analysis for IL‐17 of periapical lesions on day 21 after pulp exposure. *n* = 3.  ^∗^ *p* < 0.05,  ^∗∗^ *p* < 0.01,  ^∗∗∗^ *p* < 0.001,  ^∗∗∗∗^ *p* < 0.0001. The statistically significant *p*‐value was set at *p* < 0.05. Figure S3. IL‐17 promotes NETs formation in vitro. (A) Representative images of CitH3 (red), MPO (green), and DAPI (blue) immunolabeling of neutrophils. The white arrows indicated the coexpression regions. The selected boxes provided a partial enlarged view of the NETs in the stained images. The white arrows indicated the coexpression regions. Bar indicates 20 μm. (B) Quantitative analysis of the mean fluorescence intensity of CitH3. *n* = 3. (C) Quantitative analysis of the mean fluorescence intensity of MPO. *n* = 3. (D) Quantitative analysis of the colocalization of CitH3 and MPO using Pearson’s coefficient. *n* = 3.  ^∗^ *p* < 0.05,  ^∗∗^ *p* < 0.01,  ^∗∗∗^ *p* < 0.001,  ^∗∗∗∗^ *p* < 0.0001. The statistically significant *p*‐value was set at *p* < 0.05.

## Data Availability

The datasets used and/or analyzed during the current study are available from the corresponding author upon reasonable request.
