# Supplementary material for: The Dynamic Expression Changes of Neutrophil Extracellular Traps in Mouse Apical Periodontitis: A Potential Correlation With IL‐17
Source: J Immunol Res. 2025 Sep 15;2025:8039031. doi: 10.1155/jimr/8039031 (PMC12445725; doi:10.1155/jimr/8039031)
Supplement: Supplementary file 1 — Supporting Information Figure S1. Dynamic changes in neutrophil elastase (NE) and CitH3 in mice periapical lesions. (A) Representative images of NE (red) and DAPI (blue) immunolabeling of periapical lesions. Bar indicates 100 μm. (B) Quantitative analysis of the mean fluorescence intensity of NE. n = 3. (C) Western blot for CitH3 of the periapical lesions. n = 3. ∗ p < 0.05, ∗∗ p < 0.01, ∗∗∗ p < 0.001, ∗∗∗∗ p < 0.0001. The statistically significant p‐value was set at p < 0.05. Figure S2. Dynamic changes in IL‐17 in mice periapical lesions. (A) RT‐qPCR analysis for IL‐17a, IL‐6, TNF‐α and (B) ELISA analysis for IL‐17 of periapical lesions on day 21 after pulp exposure. n = 3. ∗ p < 0.05, ∗∗ p < 0.01, ∗∗∗ p < 0.001, ∗∗∗∗ p < 0.0001. The statistically significant p‐value was set at p < 0.05. Figure S3. IL‐17 promotes NETs formation in vitro. (A) Representative images of CitH3 (red), MPO (green), and DAPI (blue) immunolabeling of neutrophils. The white arrows indicated the coexpression regions. The selected boxes provided a partial enlarged view of the NETs in the stained images. The white arrows indicated the coexpression regions. Bar indicates 20 μm. (B) Quantitative analysis of the mean fluorescence intensity of CitH3. n = 3. (C) Quantitative analysis of the mean fluorescence intensity of MPO. n = 3. (D) Quantitative analysis of the colocalization of CitH3 and MPO using Pearson’s coefficient. n = 3. ∗ p < 0.05, ∗∗ p < 0.01, ∗∗∗ p < 0.001, ∗∗∗∗ p < 0.0001. The statistically significant p‐value was set at p < 0.05. [file JIMR-2025-8039031-s001.docx]

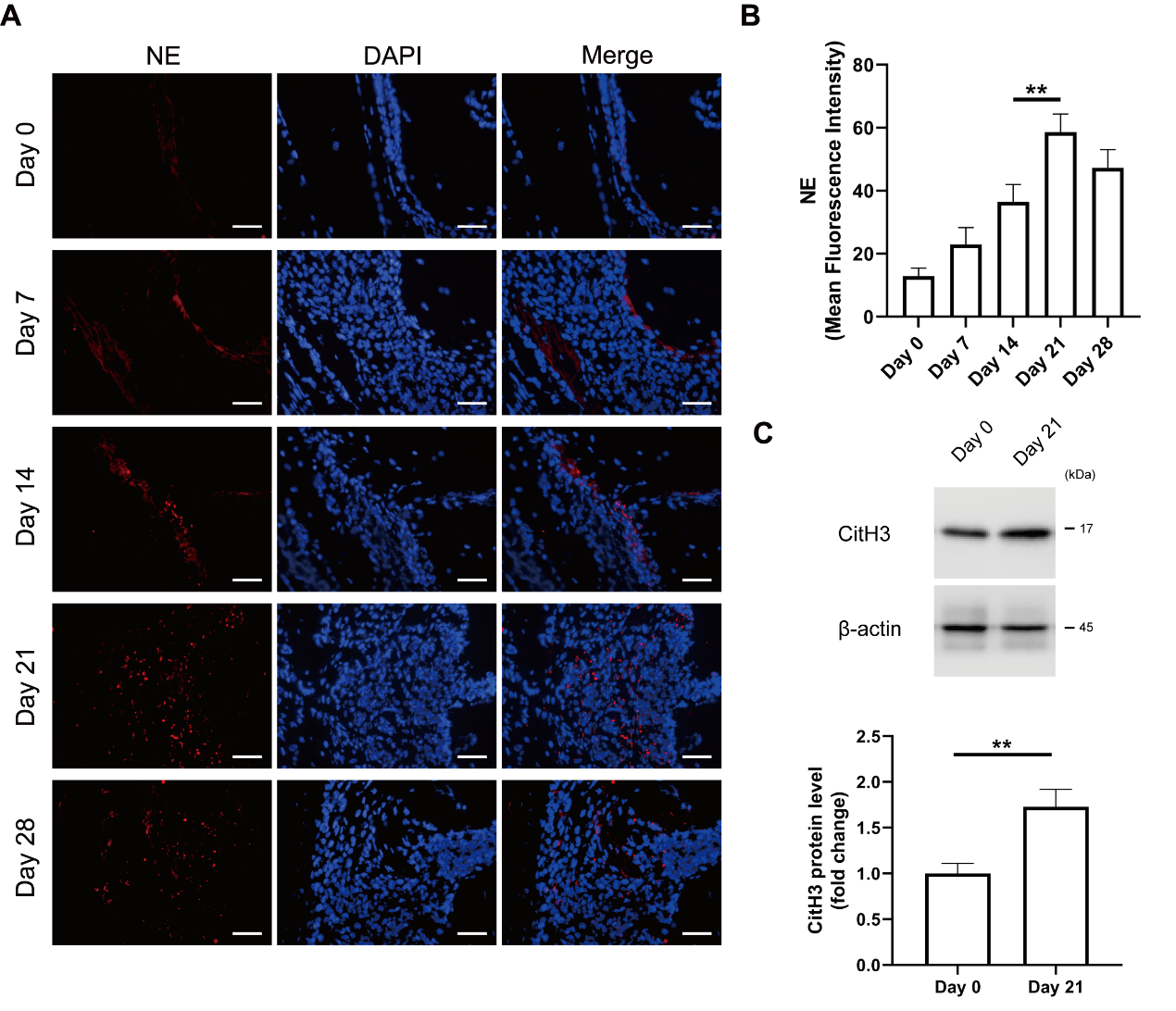


**Supplementary Fig. S1. Dynamic changes in Neutrophil elastase (NE) and CitH3 in mice periapical lesions.** (A) Representative images of NE (red) and DAPI (blue) immunolabeling of periapical lesions. Bar indicates 100 μm. (B) Quantitative analysis of mean fluorescence intensity of NE. *n*=3. (C) Western blot for CitH3 of the periapical lesions. *n*=3. **p*<0.05, ***p*<0.01, ****p*<0.001, *****p*<0.0001. The statistically significant *p* value was set at *p*<0.05.


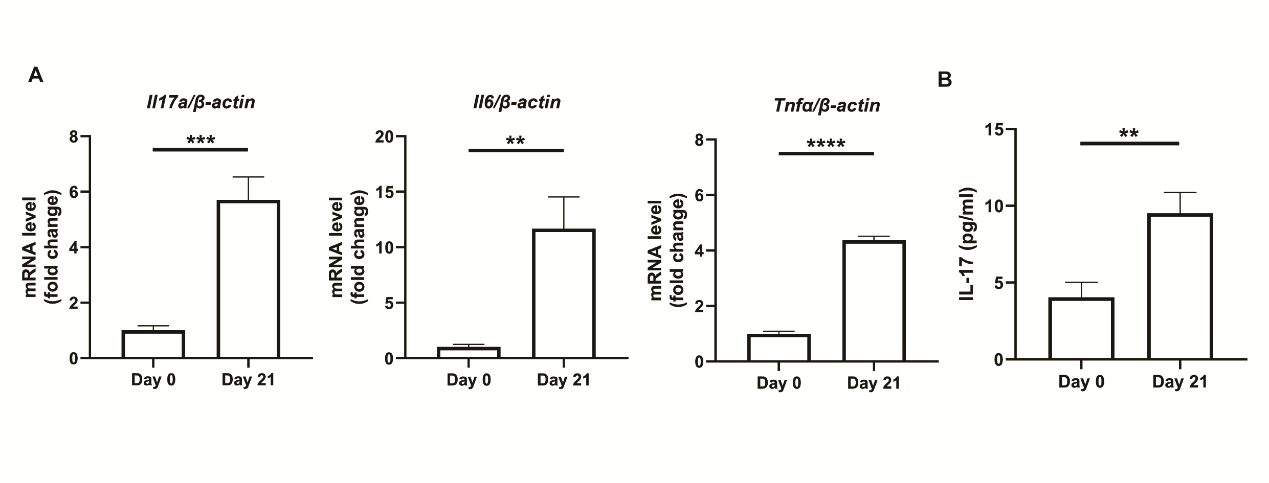


**Supplementary Fig. S2. Dynamic changes in IL-17 in mice periapical lesions.** (A) RT-qPCR analysis for *Il17a*, *Il6*, *Tnfα* and (B) ELISA analysis for IL-17 of periapical lesions on day 21 after pulp exposure. *n*=3. **p*<0.05, ***p*<0.01, ****p*<0.001, *****p*<0.0001. The statistically significant *p* value was set at *p*<0.05.

**
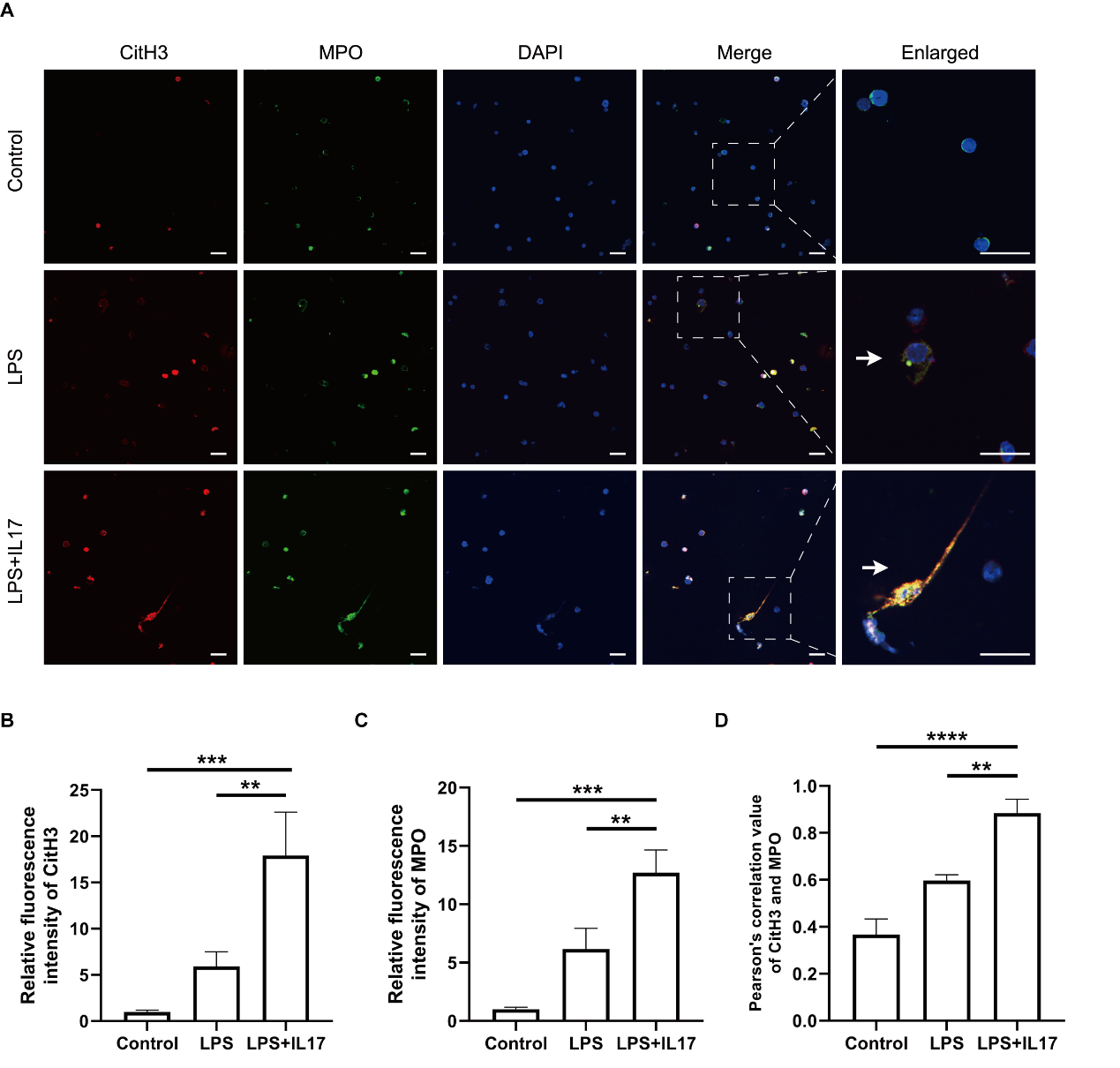
**

**Supplementary Fig. S3. IL-17 promotes NETs formation *in vitro*.** (A) Representative images of CitH3(red), MPO (green), and DAPI (blue) immunolabeling of neutrophils. The white arrows indicated the co-expression regions. The selected boxes provided a partial enlarged view of the NETs in the stained images. The white arrows indicated the co-expression regions. Bar indicates 20 μm. (B) Quantitative analysis of mean fluorescence intensity of CitH3. *n*=3. (C) Quantitative analysis of mean fluorescence intensity of MPO. *n*=3. (D) Quantitative analysis of the colocalization of CitH3 and MPO using Pearson's coefficient. n=3. **p*<0.05, ***p*<0.01, ****p*<0.001, *****p*<0.0001. The statistically significant *p* value was set at *p*<0.05.
